# Encephalitis-like presentation of methylmalonic acidemia with homocystinuria in a postpartum woman: a case report

**DOI:** 10.3389/fpsyt.2026.1734901

**Published:** 2026-03-11

**Authors:** Qianqian Wang, Zhongmin Ji, Yuzhong Wang, Ziyou Qi

**Affiliations:** 1 Department of Neurology, Affiliated Hospital of Jining Medical University, Jining, China; 2 Clinical Medicine Department, Affiliated Hospital of Jining Medical University, Jining, China

**Keywords:** encephalopathy, hyperhomocysteinemia, metabolism of cobalamin associated C gene, methylmalonic acidemia with homocystinuria, postpartum depression, postpartum period

## Abstract

Methylmalonic acidemia with homocystinuria (MMA-HC) is a rare inherited metabolic disorder characterized by diverse and nonspecific clinical manifestations. Here, we report the first case of MMA-HC presenting in the postpartum period, aiming to enhance clinicians’ awareness and diagnostic capabilities regarding this condition. This will help prevent misdiagnosis and missed diagnosis, thereby enabling timely and effective treatment for patients. A 17-year-old woman who underwent cesarean section presented with encephalitis-like symptoms shortly after childbirth, including fever, headache, psychiatric disturbances, and limb weakness, accompanied by a progressive macrocytic anemia that worsened significantly from the late prenatal to the early postpartum period, along with markedly elevated red cell distribution width (RDW) and leukopenia and severe hyperhomocysteinemia. Genetic testing confirmed cblC-type MMA-HC. A metabolic crisis was triggered after administering valproate for seizures. The patient was effectively treated with a multidisciplinary approach, highlighting the importance of careful clinical monitoring and systematic metabolic screening in peripartum women presenting with progressive hematological abnormalities and encephalopathic symptoms. It further validates the critical role of early diagnosis and multidisciplinary comprehensive treatment in managing complex inherited metabolic disorders, thereby contributing to enhanced overall diagnostic and therapeutic standards.

## 1 Introduction

Methylmalonic Acidemia with Homocystinuria (MMA-HC) is an autosomal recessive disorder affecting intracellular cobalamin metabolism, primarily caused by biallelic pathogenic variants in the Metabolism of Cobalamin Associated C (MMACHC) gene. This defect hampers the production of both adenosylcobalamin and methylcobalamin, leading to the buildup of methylmalonic acid and homocysteine (1). This autosomal recessive disorder interferes with the production of adenosylcobalamin and methylcobalamin, resulting in toxic buildup of methylmalonic acid (MMA) and homocysteine. The estimated incidence of MMA in different countries worldwide ranges from 1 in 48,000 to 1 in 250,000 (2). Adult-onset cases are often overlooked because of their nonspecific symptoms. Physiological stressors, such as pregnancy, infection, or increased metabolic demands, frequently reveal hidden cases (3). Delayed diagnosis remains a significant challenge, requiring increased clinical suspicion in adults with unexplained neurological, psychiatric, or multisystemic symptoms. A definitive diagnosis depends on a combination of biochemical tests (such as elevated plasma homocysteine and MMA, along with low cobalamin) and genetic confirmation of MMACHC variants (4). Recent studies highlight pregnancy as a significant trigger for metabolic decompensation in women with MMA-HC deficiency (5). The additional metabolic stress of gestation may overload compromised cobalamin-dependent pathways, leading to serious complications like hyperhomocysteinemia-induced encephalopathy. Variants in the MMACHC gene impair the production of two key coenzymes in vitamin B12 metabolism: adenosylcobalamin (cofactor for methylmalonyl-CoA mutase) and methylcobalamin (cofactor for methionine synthase) (6). Deficiencies in these cofactors lead to elevated serum and urinary homocysteine and methylmalonic acid levels, accompanied by correspondingly decreased methionine levels. Most early-onset patients have a poor prognosis, while late-onset cases primarily manifest as neurological degeneration accompanied by systemic symptoms. However, common physiological changes during pregnancy, such as mild anemia and fatigue, may overlap with MMA-HC symptoms, complicating clinical differentiation. Therefore, MMA-HC should be strongly suspected in pregnant or postpartum women presenting with unexplained neurological, psychiatric, or multisystem symptoms. Systematic metabolic screening and genetic testing are essential for definitive diagnosis. Here, we present the first reported case of MMA-HC manifesting in the postpartum period, suggesting a close association between pregnancy and MMA-HC onset. This provides clinicians with additional diagnostic insights and differential diagnostic criteria, enabling timely and effective treatment for patients and improving their prognosis.

## 2 Case presentation

**Figure 1:**
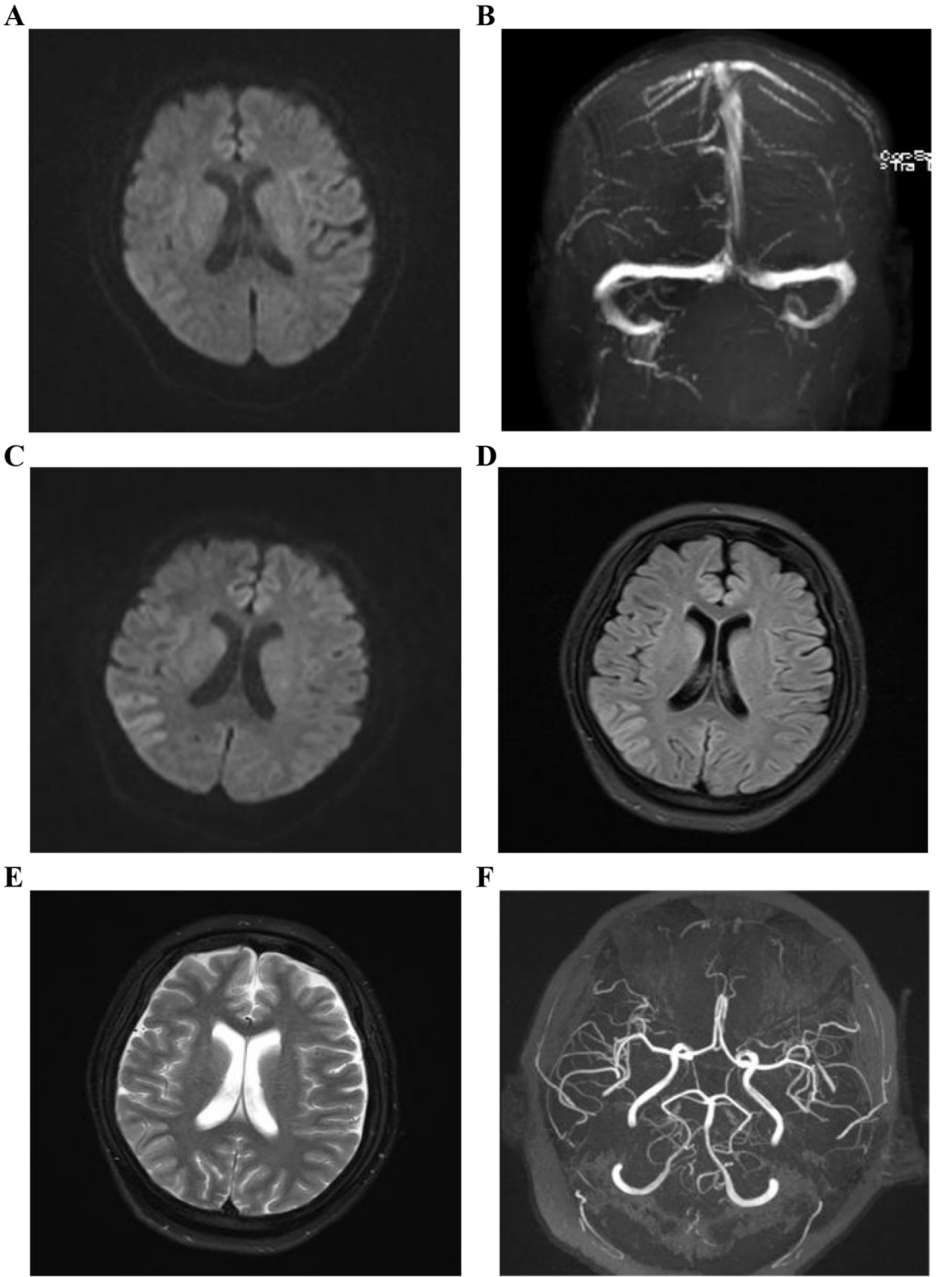
Magnetic resonance images of the patient after the onset of symptoms. **(A, B)** DWI and MRV showed no abnormalities on the ninth day of illness. DWI, diffusion weighted imaging; MRV, monitoring reporting verification; **(C-F)** MRI and MRA taken on day 14 of illness showed mild swelling of the right temporoparietal cortex with DWI hyperintensity and normal vascular findings, respectively. MRI, magnetic resonance imaging; MRA, magnetic resonance angiography; DWI, diffusion weighted imaging.

**Figure 2:**
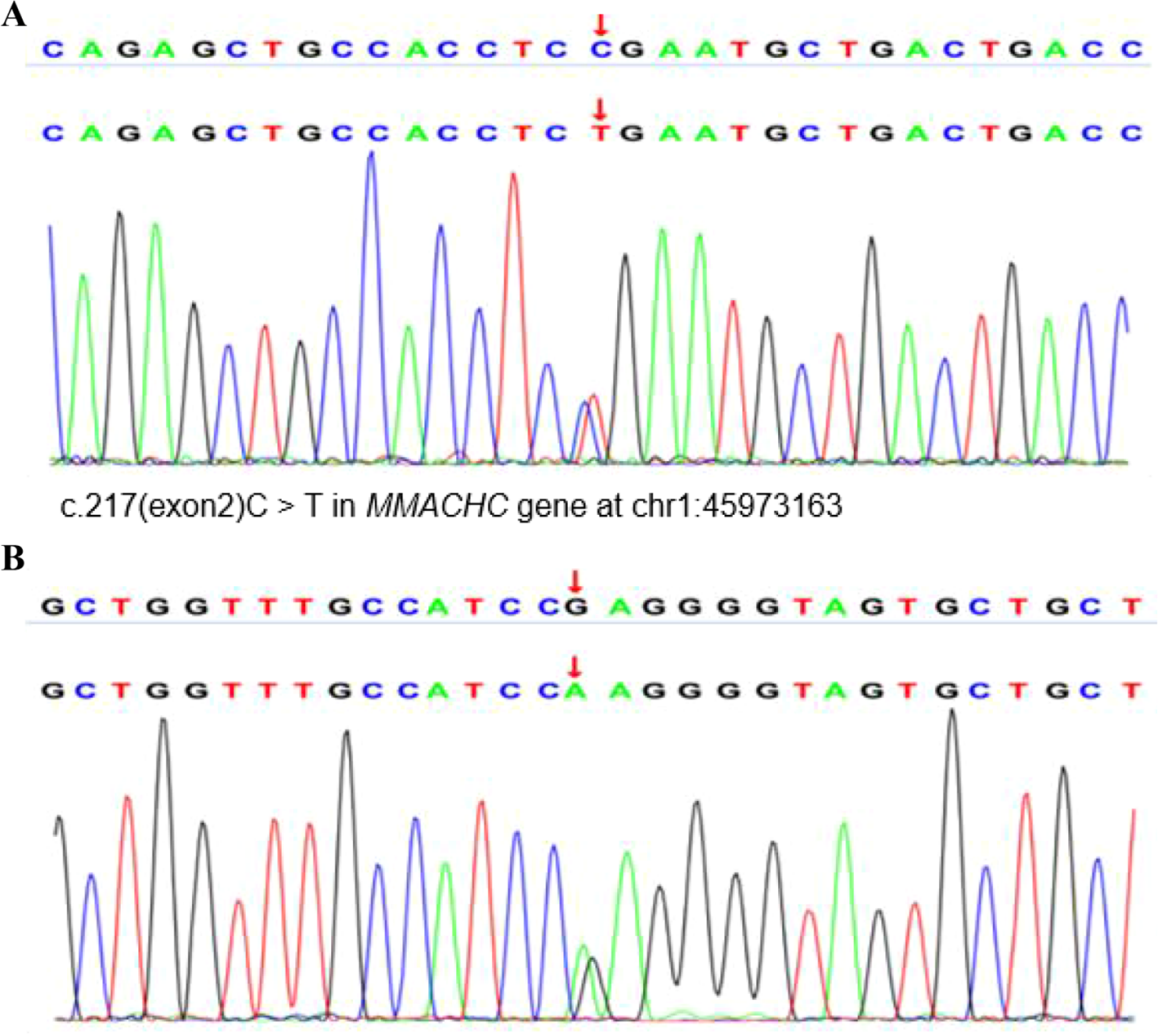
The diagnosis of methylmalonic aciduria and homocystinuria (cblC type) involves detecting compound-heterozygous pathogenic variants in the MMACHC gene. **(A, B)** Whole exome sequencing revealed a nonsense mutation (c.217C > T; p. Arg73Ter) in exon two and a missense mutation (c.482G > A; p. Arg161Gln) in exon 4. MMACHC, Metabolism of Cobalamin Associated C.

A 17-year-old woman, primigravida and with obesity (BMI: 31.2 kg/m²), was admitted to Neurology with an 8-day history of lethargy, weakness in both lower limbs, numbness, and neuropsychiatric symptoms, including slurred speech and cognitive difficulties. Initial anemia was first identified at 31 weeks and 1 day of gestation, with a hemoglobin (Hb) level of 106 g/L, indicating mild anemia. The patient reported no specific discomfort symptoms. On the day prior to delivery (at 38 weeks and 1 day), her Hb had further decreased to 99 g/L, and laboratory tests revealed macrocytosis (mean corpuscular volume 96.7 fL) and elevated red cell distribution width (RDW-SD 57.4 fL, RDW-CV 17.0%) and leukopenia (white blood cell count 2.32×10^9^/L). On day 9 of her illness (at 39 weeks gestation), magnetic resonance angiography revealed no abnormalities (**Figures 1A-B**), she had an uncomplicated cesarean section, delivering a healthy male infant (birth weight: 3,290 g; Apgar score at 1 minute: 10). During her pregnancy, she was treated for mild megaloblastic anemia with oral folate (0.8 mg/day). Her father has type 2 diabetes, and her parents are not related. On postpartum day 1, she showed delayed responses, communication problems, limb weakness, and headache. Vital signs on admission were: Blood Pressure 132/82 mmHg, Pulse 93 bpm, Respiratory Rate 20 breaths/min, and SpO_2_ 99%. Five days later, she developed a fever (38.5 °C) and severe anemia (Hb: 66 g/L), requiring a transfusion. Post-transfusion hemoglobin improved to 84 g/L. Neurological exam revealed grade 3/5 muscle strength in all limbs and generally decreased reflexes. Initial lab tests indicated severe hyperhomocysteinemia (> 150 μmol/L), elevated D-dimer (4.42 µg/mL), liver dysfunction (total bilirubin 51.7 µmol/L, direct bilirubin 26.3 µmol/L, alkaline phosphatase: 270.9 U/L), and hypoalbuminemia (Total protein 59.4 g/L, albumin 32.1 g/L). Other key laboratory findings included a significantly elevated C-reactive protein (52.5 mg/L), negative blood cultures and normal renal function tests (creatinine 50.4 µmol/L, urea 6.05 mmol/L), and elevated uric acid (406.5 µmol/L) and urinalysis showing leukocyte esterase 3+, occult blood 3+, and protein 1+ with confirmed Candida glabrata infection on culture. Cerebro-Spinal Fluid (CSF) analysis showed mild hypoglycorrhachia and increased lactate dehydrogenase. Electroencephalogram (EEG) revealed diffuse slowing with sharp wave activity. Magnetic resonance imaging (MRI) showed mild swelling of the right parietotemporal cortex (**Figures 1C-F**). Nerve conduction studies indicated polyneuropathy and facial nerve involvement. Her condition worsened on day 6, marked by the appearance of left-limb myoclonus, unresponsiveness, tachycardia, hypoglycemia, and elevated ammonia levels (39.7 μmol/L). An EEG indicated widespread cerebral slowing, while an MRI showed edema in the right parietotemporal cortex. Valproate sodium was started due to suspected seizures, but this was followed by a quick decline in consciousness and a significant deterioration of metabolic conditions, leading to a severe metabolic crisis. A diagnostic breakthrough occurred when metabolic screening of her infant indicated a severe carnitine deficiency. Valproate was immediately stopped because of its known inhibitory effect on carnitine uptake and its potential to cause hyperammonemia. Levetiracetam was used as a replacement for seizure control, and intramuscular L-carnitine (100 mg/kg/day) was started. The patient regained consciousness shortly afterward. Confirmatory tests showed severely decreased free carnitine, increased methylmalonic acid, and compound heterozygous pathogenic variants in the MMACHC gene (c.217C>T; c.482G>A), confirming cblC deficiency (**Figure 2**). Treatment with hydroxocobalamin (1 mg/week) and folate (5 mg/day) was added.

Blood and urine metabolic profiles revealed a severe deficiency of carnitine, along with elevated methylmalonic acid levels (81.2 μmol/L) and 3-hydroxybutyric acid. Whole-exome sequencing identified compound heterozygous pathogenic variants in the MMACHC gene (c.217C>T and c.482G>A), confirming a diagnosis of cblC-type methylmalonic acidemia homocystinuria.

At discharge, neuropsychiatric symptoms had partially resolved, motor function improved, and biochemical parameters normalized. By 6 months, she had achieved full neurological recovery except for a mild gait disturbance.

## 3 Discussion

This case highlights the diagnostic challenge of late-onset methylmalonic acidemia with homocystinuria (MMA-HC, cblC type) in postpartum women, a group rarely documented in the literature. The patient’s symptoms—acute encephalopathy, elevated homocysteine levels (> 150 μmol/L), and liver dysfunction—initially resembled common postpartum issues such as sepsis or blood clots, which delayed diagnosis. Hypertensive disorders of pregnancy were excluded due to normotensive readings and the absence of end-organ dysfunction. Sepsis was considered but deemed insufficient as the sole cause, given the progression of core neuropsychiatric and hematologic abnormalities despite antimicrobial therapy and negative blood cultures. Primary postpartum depression or psychosis was unlikely in the presence of objective neurological deficits and widespread metabolic derangements. Thrombotic events, particularly cerebral venous sinus thrombosis, were ruled out by normal MRV findings. This emphasizes the importance of combining metabolic screening (e.g., homocysteine, methylmalonic acid) with quick genetic testing (MMACHC analysis) in postpartum women with unexplained neuropsychiatric symptoms, especially under physiological stress like pregnancy, which can reveal underlying inherited metabolic conditions disorders (3, 4).

This case represents a true postpartum-onset metabolic crisis rather than merely pregnancy-triggered disease with postpartum recognition. The patient was largely asymptomatic with only mild, non-progressive anemia during pregnancy, which is common and non-specific. The dramatic decompensation—characterized by plummeting hemoglobin (from 99 g/L pre-delivery to 66 g/L postpartum), worsening macrocytosis and RDW, leukopenia, and the onset of severe neuropsychiatric symptoms—occurred abruptly after delivery. This timing aligns with the immense metabolic shift, protein catabolism, and hormonal changes of the puerperium, which acted as the definitive trigger for a latent metabolic disorder, making the postpartum period the true point of onset for the life-threatening manifestations.

This diagnostic odyssey underscores a critical clinical insight: the concurrence of hematological abnormalities (e.g., progressive macrocytic anemia with elevated RDW), metabolic perturbations, and encephalopathic features in a peripartum woman constitutes a powerful indication for immediate metabolic screening. Specifically, unexplained or atypical findings on routine complete blood count should prompt clinicians to measure plasma homocysteine and methylmalonic acid levels, as these readily available tests can provide the first clue to an underlying inborn error of metabolism.

The pathophysiology of cblC deficiency involves impaired intracellular cobalamin metabolism caused by MMACHC mutations, which result in toxic buildup of MMA and homocysteine (5, 7). This patient’s severe hyperhomocysteinemia probably played a role in causing endothelial dysfunction and thrombotic microangiopathy, indicated by elevated D-dimer levels and liver injury. The temporary anemia worsening after childbirth might be due to heightened metabolic demands in pregnancy overwhelming the already impaired cobalamin-dependent pathways, a pattern seen previously in women with MMA-HC during pregnancy stress (8, 9).

A key diagnostic challenge was differentiating MMA-HC from primary carnitine deficiency because of overlapping features such as hypoglycemia and hyperammonemia. Although the infant’s low free carnitine initially indicated maternal carnitine deficiency, the identification of compound heterozygous MMACHC variants (c.217C>T and c.482G>A) confirmed cblC-type MMA-HC (10). The c.482G>A mutation is strongly associated with late-onset cases (11), highlighting the need to combine genetic and metabolic profiling to resolve diagnostic ambiguity. In our case, the combination of a nonsense mutation (c.217C>T) and a missense mutation (c.482G>A) probably caused severe enzymatic dysfunction, contrasting with the milder phenotypes seen in patients with only missense mutations (3, 12). Enhanced metabolic screening during pregnancy is vital, since women with MMA are at increased risk of preterm delivery (38.5%) and cesarean section (53.8%) (4). A multidisciplinary approach involving metabolic specialists and obstetric teams is crucial to reduce these risks.

Valproate use in this case worsened metabolic instability through three mechanisms: suppression of carnitine synthesis, increased urinary excretion, and disruption of mitochondrial β-oxidation (11). This compounded the underlying defect, leading to more severe hypoglycemia (3.0 mmol/L) and hyperammonemia (39.7 μmol/L). These iatrogenic risks emphasize the importance of avoiding valproate in suspected metabolic disorders and opting for safer agents like levetiracetam (5, 9).

Therapeutic intervention using hydroxocobalamin, L-carnitine, and folate follows current guidelines. Hydroxocobalamin helps lower homocysteine levels by restoring methionine synthase activity, while L-carnitine alleviates secondary deficiencies caused by MMA accumulation (13). Although biochemical markers improve, lingering neurological issues, such as limb weakness, indicate irreversible neuronal damage resulting from prolonged hyperhomocysteinemia (14), highlighting the importance of early treatment (15).

Limitations of this case report include its nature as a single observation, which limits generalizability. The diagnosis was also made retrospectively after a severe crisis, highlighting the current lack of routine prenatal or early postpartum screening protocols for such disorders.

## 4 Conclusions

This case highlights a critical diagnostic pathway for postpartum neuropsychiatric presentations. When encephalopathy coincides with unexplained macrocytic anemia (especially with elevated RDW) in a peripartum woman, immediate metabolic screening—specifically plasma homocysteine and methylmalonic acid testing—is imperative. This screening should be triggered by: (1) refractory prenatal anemia, (2) any postpartum psychiatric/neurological symptom, and (3) blood counts suggesting megaloblastic changes, such as macrocytosis (elevated MCV) combined with elevated RDW and leukopenia, which are early indicators of metabolic crisis. These findings should prompt screening at any point during pregnancy or postpartum.

Future efforts should aim to standardize metabolic screening protocols during pregnancy, such as amniotic fluid MMA and homocysteine analysis. Additionally, fostering collaboration between obstetricians and metabolic specialists is essential. Exploring targeted therapies, including mRNA-based treatments, is also recommended (14). Clinicians need to stay vigilant for rare metabolic disorders in unusual postpartum cases to facilitate early diagnosis and better outcomes.

## Data Availability

The original contributions presented in the study are included in the article/supplementary material. Further inquiries can be directed to the corresponding author.
